# Hormetic Effects of Cerium, Lanthanum and Their Combination at Sub-micromolar Concentrations in Sea Urchin Sperm

**DOI:** 10.1007/s00128-023-03701-z

**Published:** 2023-03-15

**Authors:** Giovanni Pagano, Antonios Apostolos Brouziotis, Daniel Lyons, Ivana Čarapar, Rahime Oral, Serkan Tez, Philippe J. Thomas, Franca Tommasi, Giovanni Libralato, Marco Guida, Marco Trifuoggi

**Affiliations:** 1 Department of Chemical Sciences, Federico II Naples University, I-80126 Naples, Italy; 2 Department of Biology, Federico II Naples University, I-80126 Naples, Italy; 3 Center for Marine Research, Ruđer Bošković Institute, HR-52210 Rovinj, Croatia; 4 Faculty of Fisheries, Ege University, Bornova, TR-35100 İzmir, Turkey; 5 Environment and Climate Change Canada, Science & Technology Branch, National Wildlife Research Center, Carleton University, K1A 0H3 Ottawa, ON Canada; 6 Department of Biology, “Aldo Moro” Bari University, I-70125 Bari, Italy

**Keywords:** Rare earth elements, Fertilization, Developmental defects, Transmissible effects, Hormesis

## Abstract

Rare earth elements (REEs) cerium (Ce) and lanthanum (La) and their combination were tested across a concentration range, from toxic (10^−4^ to 10^−5^ M) to lower concentrations (10^−6^ to 10^−8^ M) for their effects on sea urchin *(Sphaerechinus granularis)* sperm. A significantly decreased fertilization rate (FR) was found for sperm exposed to 10^−5^ M Ce, La and their combination, opposed to a significant increase of FR following 10^−7^ and 10^−8^ M REE sperm exposure. The offspring of REE-exposed sperm showed significantly increased developmental defects following sperm exposure to 10^−5^ M REEs vs. untreated controls, while exposure to 10^−7^ and 10^−8^ M REEs resulted in significantly decreased rates of developmental defects. Both of observed effects–on sperm fertilization success and on offspring quality–were closely exerted by Ce or La or their combination.

## Introduction

Rare earth elements (REEs) include a group of elements, the lanthanoids [lanthanum (La) to lutetium (Lu)] and two closely related elements, yttrium (Y) and scandium (Sc) which are recognized to be indispensable in the present world, due to their extensive roles in a number of technologies (Du and Graedel 2013; Pagano et al. 2015; González et al. 2015). REE-associated adverse effects have been assessed in a vast body of literature encompassing several biota, with implications also in human health, so that REEs have raised extensive health concern and are viewed as emergent contaminants (Brouziotis et al. 2022; Gravina et al. 2018; Thomas et al. 2014; Trifuoggi et al. 2017).

Apart from their adverse effects, REEs also display recognized stimulatory effects, as reported in a body of literature on their use as components of fertilizers improving crop yields and in livestock feed additives (Abdelnour et al. 2019; Agathokleous et al. 2019; Bölükbaşı et al. 2016; He et al. 2010; Lian et al. 2019; Tommasi et al. 2021; Yin et al. 2021; Zhang et al. 2018). This duplicity of REE-associated effects is not specific for REEs, but may be ascribed to a general phenomenon of a concentration-related shift from inhibition, or “toxicity” for high agent concentrations to stimulation for lower agent concentrations, also termed hormesis, and previously tagged as “Arndt-Schulz effect” (Stebbing 1982; Pagano et al. 1982; Cedergreen et al. 2006; Agathokleous et al. 2020; Calabrese 2016; Técher et al. 2020). The multiple implications of hormesis have been reported in an extensive body of basic and applied disciplines (e.g. Agathokleous et al. 2022; Calabrese et al. 2022; Katsnelson et al. 2021; Lee and Lee, 2019; Jalal et al. 2021; Nitti et al. 2022; Schirrmacher, 2021; Shibamoto and Nakamura, 2018).

Within the frame of REE-associated hormetic effects, the present study was aimed at verifying the effects of micromolar and sub-micromolar levels of two REEs, Ce and La, and their combination on sea urchin sperm fertilization success and offspring embryogenesis. The results confirmed a shift from inhibition to stimulation of tested events by comparing 10^−5^ M vs. < 10^−6^ M.

## Materials and Methods

Cerium nitrate, lanthanum nitrate and their equimolar combinations were tested for their effects on *Sphaerechinus granularis* sea urchin sperm in changing fertilization success and the frequency of developmental defects in the offspring of exposed sperm. A preliminary assay tested a duration of control sperm suspension (10 to 60 min), allowing to either assess inhibition or stimulation of fertilization rate, leading to an intermediate (∼50%) fertilization rate, and was found as 30-min sperm exposure (Pagano et al. 2017).

Sperm suspension was carried out by 1% dilution of “dry” sperm (as released by testes from two males) in agent solutions at concentrations ranging from 10^−8^ to 10^−5^ M. These duplicate sperm suspensions, in turn, fertilized eggs from three females, thus providing six-replicate embryo cultures that were observed for fertilization rate (FR, % fertilized eggs) and then for offspring quality. FR was measured starting from the appearance of fertilization membrane and of early cleavage (2-cell stage) for approximately 3 h post-fertilization. Subsequent observation of offspring was performed 3 days post-fertilization allowing detection of % prepared immediately before analysis developmental defects (DD) as larval malformations or pre-larval arrest and of mortality. This observation was carried out after immobilizing larvae and embryos by adding a 10^−4^ M chromium sulfate, which allowed screening of bottom-laying embryos/larvae in an inverted microscope, ×10 magnification.

Analytical concentrations of Ce and La in the samples were determined by inductively coupled plasma mass spectrometry (ICP-MS, Aurora M90 Bruker, Germany). A Milli-Q unit (Millipore, United States) was used to obtain high-purity water (resistivity = 18.2 MΩ cm) was obtained from a Milli-Q unit (Millipore, United States). Nitric acid (HNO_3_, 69% v/v Ultratrace@ ppb-trace analysis grade) was purchased from Scharlau (Barcelona, Spain). All samples analyzed in ICP-MS were prepared in HNO_3_ solution (2% v/v). The analysis was performed in High Sensitivity mode. Calibration curves for determining REEs ranged from 0.5 to 1,000 µg/L for Normal and from 0.005 to 10 µg/L for High Sensitivity and were constructed daily by analysis of standard solutions prepared immediately before analysis. The internal standard was ^115^In for both calibration curve and sample analysis.

The uniform and minimal weight concentration of sperm cells was not measured.

Datasets were analyzed in IBM SPSS v20 and Microsoft® Excel 2013/XLSTAT©-Pro (Version 7.2, 2003, Addinsoft, Inc., Brooklyn, NY, USA). Homogeneity of variances was checked by Levene’s test. Differences between each concentration group and the controls were determined by two-tailed Independent Samples t-test. A normality test was performed and the significance of the difference among the groups was evaluated by One-way Analysis of Variance (ANOVA). Differences were considered significant when *p* < 0.05.

## Results and Discussion

The correspondence between nominal and analytical concentrations of the tested samples, measured by ICP-MS, is shown in Table 1. The analytical/nominal ratios mostly ranged from 0.889 to 1.283; thus nominal concentration values were considered as reliable for concentration-related trends.

**Table 1:** Ratios of analytically checked concentrations of tested REEs (by ICP-MS, as µg/L) vs. nominal concentrations

Nominal concentrations	10^−4^ M	10^−5^ M	10^−6^ M	10^−7^ M	10^−8^ M
Ce	1.095	1.008	1.102	1.277	1.021
La	0.92	0.883	1.238	1.053	1.224

**Fig. 1:**
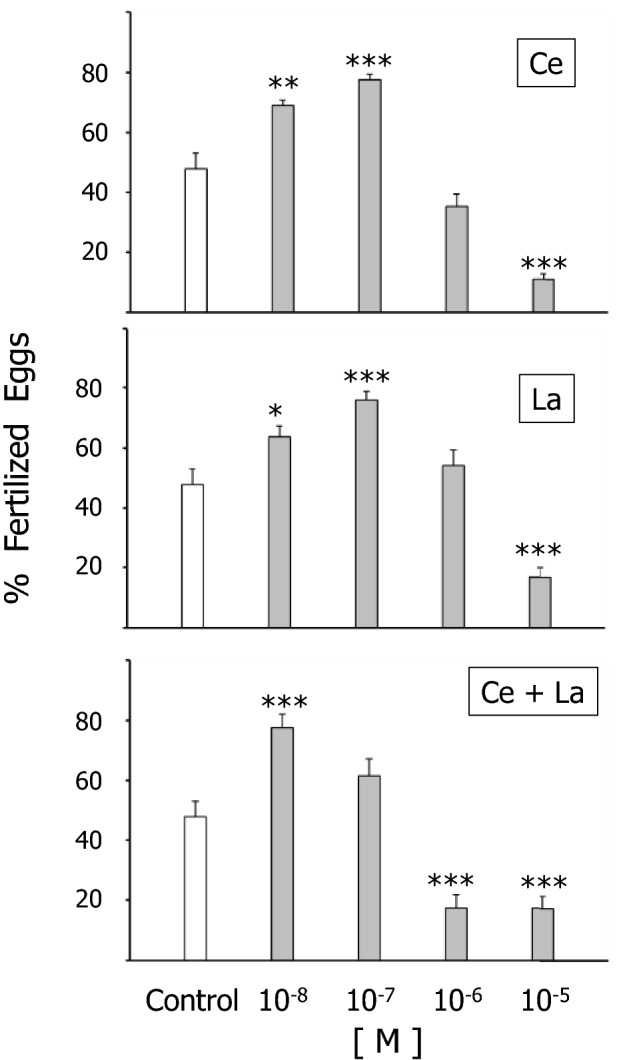
Percent fertilization rate of *S. granularis* sperm exposed 30 min to Ce(NO_3_), or La(NO_3_), or their combination at concentrations ranging from 10^−8^ to 10^−5^ M

As shown in Fig. 1, fertilization rate (FR) of *S. granularis* sperm exposed for 30 min to Ce(NO_3_)_3_, or La(NO_3_)_3_, or their equimolar combination at concentrations ranging from 10^−8^ to 10^−5^ M showed the expected spermiotoxicity following 10^−5^ M pretreatment as reported previously (Trifuoggi et al. 2017). No significant effect was detected following sperm exposure to 10^−6^ M Ce(NO_3_)_3_ or La(NO_3_)_3_ level, with a significant FR decrease was observed following sperm exposure to 10^−6^ M Ce + La combination. Lower agent levels, as 10^−7^ and 10^−8^ M Ce, La and Ce + La combination resulted in significant FR increase.

**Fig. 2:**
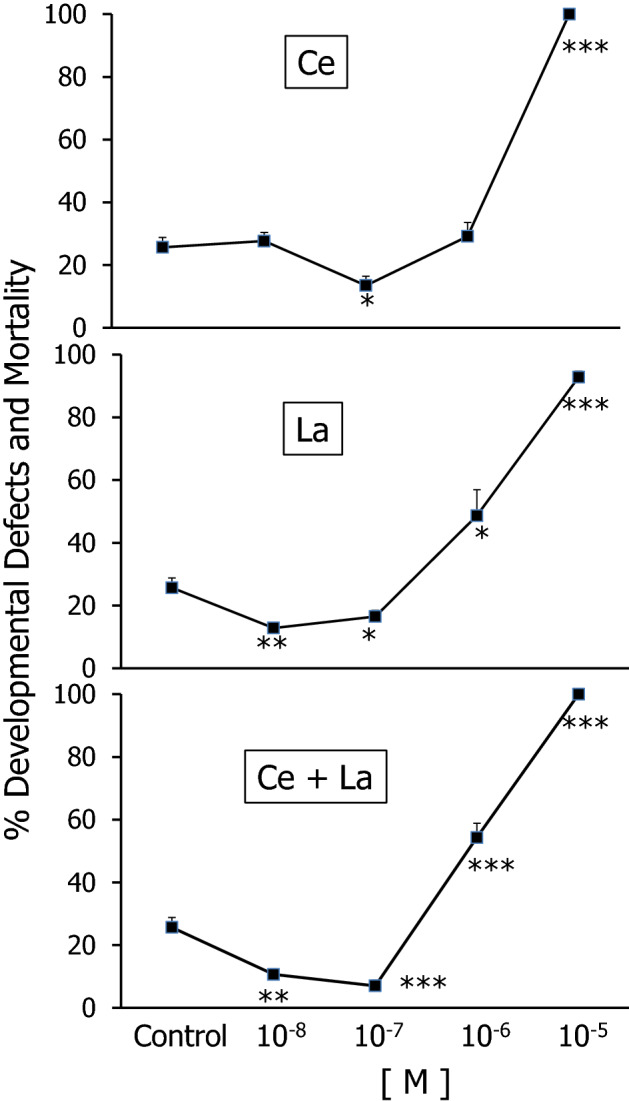
Percent developmental defects and mortality in the offspring of *S. granularis* sperm exposed 30 min to Ce(NO_3_), or La(NO_3_), or their combination at concentrations ranging from 10^−8^ to 10^−5^ M

The opposite concentration-related trend was found for the frequency of developmental defects (DD) and mortality (M) in the offspring of Ce-, La- and (Ce + La)-exposed sperm, which was significantly increased following sperm exposure to 10^−5^ M agents and to Ce 10^− 6^ M, whereas lower agent concentrations (10^−7^ and 10^−8^ M Ce, or La or their combination) in sperm exposure resulted in significantly decreased offspring DD and M, as shown in Fig. 2.

The present results confirm the established database of REE-associated spermiotoxicity and induction of offspring damage following sperm exposure to Ce and La at concentrations ≥ 10^−5^ M (Trifuoggi et al. 2017). On the other hand, this study provides evidence for a hormetic shift of these REEs and of their combination at sub-micromolar concentrations that were found to significantly increase fertilization success and to decrease offspring anomalies vs. controls. Trans-generational hormetic effects were reported by Agathokleous et al. 2022. These results are new, though they could be anticipated on the grounds of the established use of REEs in supporting animal growth (He et al. 2010; Bölükbaşı et al. 2016; Abdelnour et al. 2019). Should these results be further confirmed in other bioassay models, they provide the grounds toward REE utilization in safely promoting animal growth.
